# Antibodies to Full-Length Agrin Protein in Chinese Patients With Myasthenia Gravis

**DOI:** 10.3389/fimmu.2021.753247

**Published:** 2021-12-08

**Authors:** Shumin Wang, Haonan Yang, Rongjing Guo, Lulu Wang, Yingna Zhang, Jie Lv, Xue Zhao, Jing Zhang, Hua Fang, Qingyong Zhang, Yunke Zhang, Junhong Yang, Xinzheng Cui, Peiyang Gao, Ting Chang, Feng Gao

**Affiliations:** ^1^ Department of Neuroimmunology, Henan Institute of Medical and Pharmaceutical Sciences, Academy of Medical Science, Zhengzhou University, Zhengzhou, China; ^2^ BGI College, Zhengzhou University, Zhengzhou, China; ^3^ Department of Neurology, Tangdu Hospital, The Air Force Medical University, Xi’an, China; ^4^ Department of Neurology, The Second Affiliated Hospital of Zhengzhou University, Zhengzhou, China; ^5^ Myasthenia Gravis Comprehensive Diagnosis and Treatment Center, Henan Provincial People’s Hospital, Zhengzhou, China; ^6^ Department of Encephalopathy, First Affiliated Hospital of Henan University of Traditional Chinese Medicine (TCM), Zhengzhou, China

**Keywords:** myasthenia gravis, cell-based assay, agrin, autoantibody, clinical features

## Abstract

This study aimed to establish a cell-based assay (CBA) for the detection of agrin antibodies (Agrin-Ab) to explore the clinical features of agrin antibody-positive Chinese patients with myasthenia gravis (Agrin-MG). We developed a CBA based on the human full-length agrin protein expressed in HEK293T cells for the reliable and efficient detection of Agrin-Ab. Clinical data and serum samples were collected from 1948 MG patients in 26 provinces in China. The demographic and clinical features of Agrin-MG patients were compared with those of other MG patient subsets. Eighteen Agrin-MG cases were identified from 1948 MG patients. Nine patients were Agrin-Ab positive, and nine were AChR-Ab and Agrin-Ab double-positive (Agrin/AChR-MG). Eleven (61.11%) patients were males older than 40 years of age. The initial symptom in 13 (81.25%) cases was ocular weakness. Occasionally, the initial symptom was limb-girdle weakness (two cases) or bulbar muscle weakness (one case). Agrin-MG patients demonstrated slight improvement following treatment with either acetylcholinesterase inhibitor or prednisone; however, the combination of the two drugs could effectively relieve MG symptoms. In China, Agrin-MG demonstrated seropositivity rates of 0.92%. These patients were commonly middle-aged or elderly men. The patients usually presented weakness in the ocular, bulbar, and limb muscles, which may be combined with thymoma. These patients have more severe diseases, although the combination of pyridostigmine and prednisone was usually effective in relieving symptoms.

## 1 Introduction

Myasthenia gravis (MG) is an autoimmune disease characterized by partial or systemic skeletal muscle weakness and fatigue (1, 2). The incidence rate of MG is 0.3–2.8 per 100,000 people per year worldwide (3, 4) and 0.68 per 100,000 people per year in China (5). About 80% of MG cases are caused by autoantibodies against the acetylcholine receptor (AChR-Ab) (6, 7), these patients are known as AChR-MG. Around 20–50% of AChR-Ab negative patients have autoantibodies against muscle-specific tyrosine receptor kinase (MuSK-Ab) (8–10), known as MuSK-MG. Among patients who are double negative for AChR-Ab and MuSK-Ab, 2–19% are positive for low-density lipoprotein receptor-related protein 4 antibody (LRP4-Ab) (11, 12), known as LRP4-MG.

Recent studies have shown that Agrin-Ab is a novel type of MG pathogenic antibody (13, 14). Furthermore, agrin antibodies were also detected serologically in triple-seronegative MG patients (15) (no detectable AChR, MuSK, and LRP4 autoantibodies, referred to as TSN-MG). Thus far, Agrin-MG patients have not been reported in China. Because only a few cases have been detected worldwide, Agrin-MG patients’ clinical and demographic characteristics have not been reported.

**Figure 1:**
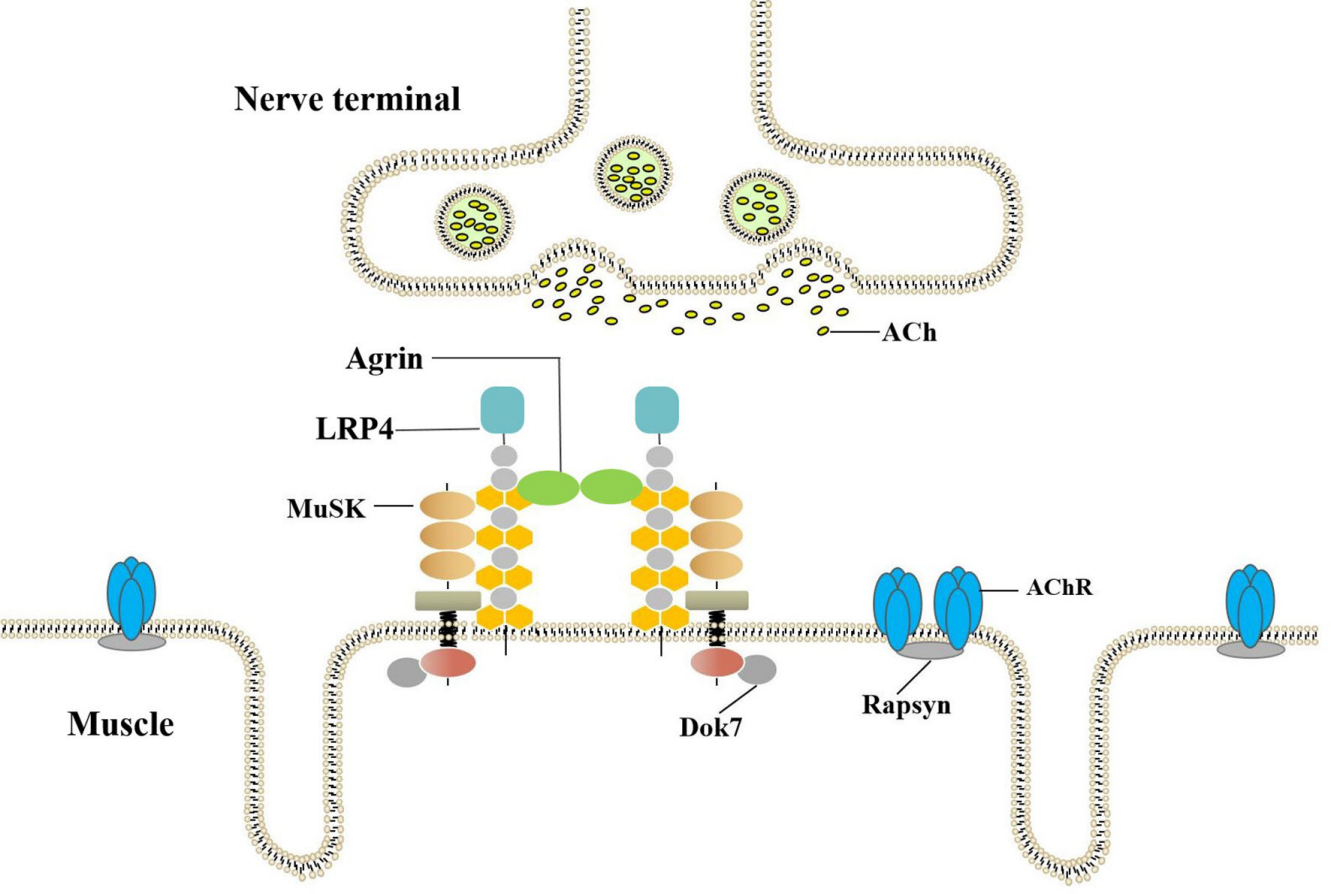
Agrin-LRP4-MuSK-Dok7 pathway for AChR clustering. The combination of Agrin and LRP4 activates MuSK. Recruitment of Dok-7 further enhances MuSK dimerization resulting in the full activation of the MuSK kinase. The MuSK signaling then induces AChR aggregation in postsynaptic.

The *AGRN* gene is divided into two tissue-specific subtypes, namely, muscle-agrin (M-agrin) and neural agrin (N-agrin). M-agrin lacks any z insert and may be involved in endothelial cell differentiation. N-agrin is a special isoform expressed in motor neurons. The neuron-specific (z+) isoforms that contain C-terminal insertions of 8-19 amino-acid are potent activators of AChR clustering. Agrin binding to Lrp4 increases MuSK-Lrp4 interaction. Upon binding, the Lrp4/MuSK tetramer presumably rearranges in a way that leads to dimerization and subsequent autophosphorylation of MuSK (16, 17). Recruitment of Dok-7 further enhances MuSK dimerization resulting in the complete activation of MuSK. MuSK signaling then induces AChR aggregation in the postsynaptic space, as shown in **Figure 1** (18). N-agrin is 1000-fold more effective in clustering AChRs *in vitro* than M-agrin (lacks the insert at Z site) and is necessary for neuromuscular junction formation. Mutations in the agrin gene can cause NMJ-related diseases, including Congenital Myasthenic Syndrome (19, 20).

Cell-based assay (CBA) and ELISA detection systems based on short agrin were established, however, short agrin contains only approximately 50% of the coding sequence of agrin. Therefore, a number of epitopes present in full-length agrin are not present in short agrin. They therefore might have missed patients with autoantibodies against the central domains of agrin. More patients with agrin seropositive MG might be identified if a full-length agrin protein is used as probe (19, 20).

In the present study, we have constructed a plasmid expressing human full-length agrin protein and established a CBA to detect Agrin-Ab. We have used this assay to examine 1948 serum samples collected from patients with MG and to explore the clinical characteristics of Agrin-MG in China. Characterization of Agrin-MG patients leads to a better understanding of the disease and enables more appropriate treatment.

## 2 Patients and Methods

### 2.1 Patients

**Figure 2:**
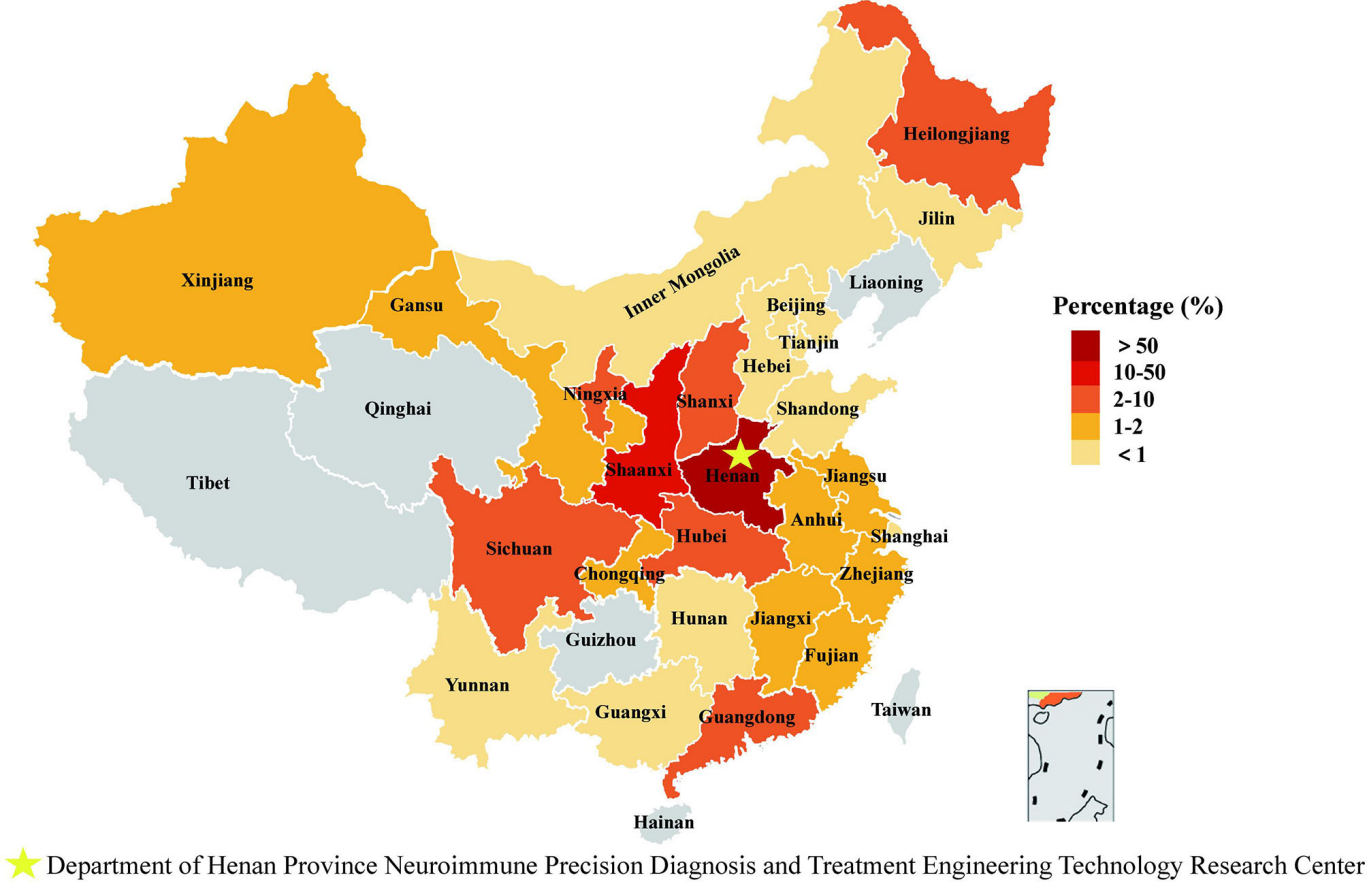
Regional distribution of specimen source. We counted the geographical origin of 1948 patients from 26 provinces/autonomous regions/municipalities in China.

From June 2017 to May 2020, 2870 serum samples from suspected MG patients were collected and tested for MG-related antibodies in the Henan Province Neuroimmune Precision Diagnosis and Treatment Engineering Technology Research Center. These samples were collected from 26 provinces in China (as shown in **Figure 2** and **Supplementary Table 1**). In total, 1948 patients were diagnosed with myasthenia gravis. A clinical diagnosis of MG was confirmed on the basis that (1) and any of (2), (3), or (4) were applicable:

(1) the clinical manifestations included typical symptoms of muscle weakness, which aggravated after activity and improved after rest;
(2) compound muscle action potential (CMAP) amplitude reduction ≥ 10% in a low-frequency repetitive nerve electrical stimulation [RNS] test;
(3) a positive neostigmine test;
(4) a positive test for MG-associated antibodies (AChR-Ab, MuSK-Ab, or LRP4-Ab).

We summarized and analyzed the clinical data of all patients diagnosed with myasthenia gravis and classified the severity of the disease according to the American Myasthenia gravis Foundation (MGFA) classification.

We assessed the serum for patients with a specific disease and positive results as follows: Lambert-Eaton myasthenic syndrome (LEMS) [(voltage-gated calcium channel; VGCC-Ab (n = 20)], idiopathic inflammatory myositis (IIM) [myositis specific autoantibody positive, MSA-Ab (n = 17); myositis associated autoantibody positive, MAA-Ab (n = 17)], peripheral neuropathy (anti-ganglioside 1 antibodies-Ab positive, GM1-Ab positive; n = 27), autoimmune encephalitis [gamma aminobutyric-acid B receptor-Ab positive (GABABR-Ab positive; n = 8) and anti-N-methyl-D-aspartate receptor-Ab positive (NMDAR-Ab positive; n = 14)] and neuromyelitis optica spectrum disorders, NMOSD [anti-aquaporin-4-Ab positive (AQP4-Ab; n = 15)], and compared them with sera from healthy controls (n = 168).

### 2.2 Methods

#### 2.2.1 Detection of Antibodies in the Serum of MG Patients

The cDNA coding human full-length neural agrin (NCBI Reference Sequence: NM 001305275.2, does not include signal peptide) was subcloned into pCMV6-AC-GFP vector (OriGene, Rockville, MD, USA), named as pCMV6-AC-Agrin-GFP. The pCMV6-AC-Agrin-GFP plasmid was used to transfect HEK293T cells (21). The expression of the full-length fusion protein was confirmed by western blotting (as shown in **Supplementary Figure 1**) and by immunofluorescence staining of transfected cells with anti-agrin antibody (DF9181, Affinity Biosciences, OH, USA).

Agrin antibody was detected using a laboratory-established CBA method. Following transfection of the pCMV6-AC-Agrin-GFP plasmid into HEK293T cells using TurboFectin 8.0, the cells were incubated for 48 h and then washed briefly with PBS. The cells were then fixed with 4% paraformaldehyde for 20 min at 4°C, washed three times with PBS, permeabilized with 0.5% Triton X-100 in PBS for 5 min, and finally blocked with 5% BSA at room temperature for 3 h. The serum was diluted 1:10 and then incubated with the permeabilized and blocked cells for 1 h at room temperature (25°C). After washing three times, the cells were incubated with Alexa Fluor-568 goat anti-human IgG antibody (A-21090, Invitrogen, CA, USA. 1:1000 diluted) at room temperature for 2 h and then washed with PBS three times. Antibodies against agrin were detected using a fluorescence microscope set up to detect red fluorescent secondary antibody binding.

The samples were scored (22) according to fluorescence intensity and the number of overlapping cells as follows: 0, no signal; 0.5 for very weak labeling of a few cells with no definite colocalization; 1, 1 for weak labeling of some cells with colocalization; 2 for labeling of 20–50% of cells with accurate colocalization, 3 for labeling of 50–80% of cells with perfect colocalization, and 4 for labeling of all transduced cells showing perfect colocalization. The scores from each serum sample were independently evaluated by two trained observers. For tests with an average score ≥ 1 and ≥ 2, the result was considered positive. An average score ≤ 0.5 was considered negative.

Agrin IgG subclass (23) was determined by incubating the serum with the cells, washing the cells, and incubating the cells with mouse anti-human IgG subclass antibodies (C010215, C010214, C010212, C010211, CELLWAYLAB, China. http://www.ablab.com.cn/; 5 mg/mL, 1:250 diluent) at room temperature for 1 h. The cells were then incubated with Alexa Fluor-594 donkey anti-mouse IgG (715-585-150, PA, USA. 1:500 dilution) at room temperature for 2 h. The samples were then processed as described above.

An AChR-Ab ELISA kit (RSR Ltd, Cardiff, UK) was used to measure AChR-Ab levels. MuSK-Ab and LRP4-Ab were tested by CBA. The full procedure has been described in previous articles (24). Titin-Ab levels were measured using a Titin-Ab ELISA kit (DLD, Hamburg, Germany).

#### 2.2.2 Statistical Analyses

IBM SPSS Statistics 21 (IBM, Armonk, New York, USA) was used for charting and statistical analysis. To validate the significance of the observed differences, we analyzed simple pairwise comparisons with the Student’s t-test.

#### 2.2.3 Standard Protocol Approvals

All clinical investigations were conducted in accordance with the principles of the Helsinki Declaration. This study was approved by the Medical Ethics Committee of Henan Medical Science Research Institute of Zhengzhou University (202002), and the patients signed informed consent forms.

## 3 Results

### 3.1 Detection of Agrin Antibody in the Serum of MG Patients

**Figure 3:**
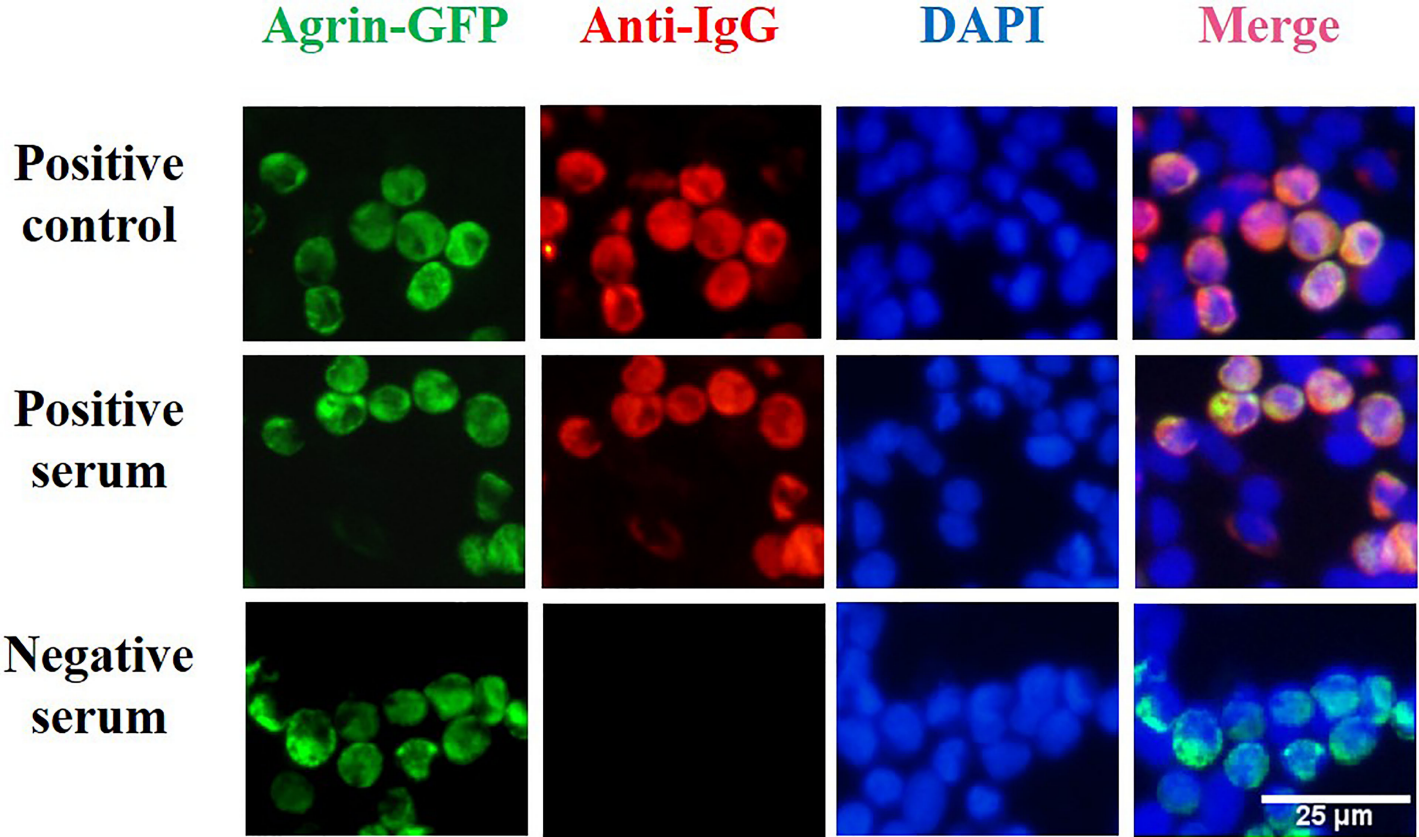
Cell-based assay for the detection of agrin antibodies. HEK293T cells were transfected with a plasmid construct encoding agrin-GFP fusion to develop a cell-based assay for the detection of agrin antibody. After transfection of HEK293T cells with the plasmid construct encoding the fusion protein, green fluorescence was used to confirm fusion protein expression in transfected cells. In our cell-based assay, the binding of IgG in the patient serum is confirmed by red fluorescence. Positive control (DF9181,Affinity Biosciences, OH, USA), HC serum, and Agrin-MG serum staining of transfected cells. The complete overlap of red fluorescence and green fluorescence indicates the presence of anti-Agrin antibodies.

HEK293T cells transfected with pCMV6-AC-Agrin-GFP were incubated with serum from MG patients, and bound human antibodies were detected with Alexa Fluor-568 goat anti-human IgG antibody (as shown in **Figure 3**). Green fluorescence was used to confirm fusion protein expression in transfected cells. The binding of IgG in the patient serum is confirmed by red fluorescence. The complete overlap of red fluorescence and green fluorescence indicates the presence of anti-Agrin antibodies. Using our CBA, we detected 18 Agrin-MG patients (using serum from 1948 MG patients). Nine patients were Agrin-Ab positive only, and nine patients were AChR/Agrin-Ab double-positive. No agrin antibody was found in 286 control samples, all of them were negative.

### 3.2 MG Antibody Frequency

**Figure 4:**
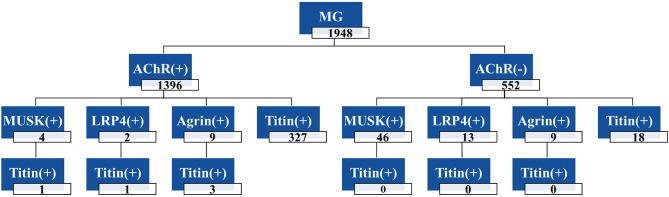
Frequencies of antibodies in 1948 MG patients. The total number of MG patients was 1948, including 1396 AChR-Ab positive patients and 552 negative patients; Four patients were double-positive for AChR-Ab and MuSK-Ab, and one patient was triple-positive for AChR-Ab, MuSK-Ab and Titin-Ab. +, positive antibody; -, negative antibody.

As shown in **Figure 4**, the frequencies of each antibody in the 1948 cases of MG patients were as follows: Agrin-Ab, 0.92%; AChR-Ab, 71.66%; MuSK-Ab, 2.56%; LRP4-Ab, 0.78%; other (AChR-Ab, MuSK-Ab, LRP4-Ab, and Agrin-Ab not detected), 24.84%. Among the 493 triple-seronegative MG patients, nine cases (1.82%) were Agrin-Ab positive. In addition, we detected nine cases of Agrin-Ab/AChR-Ab double-positive patients (three of whom were also positive for Titin antibody).

### 3.3 Demographic Characteristics

**Figure 5:**
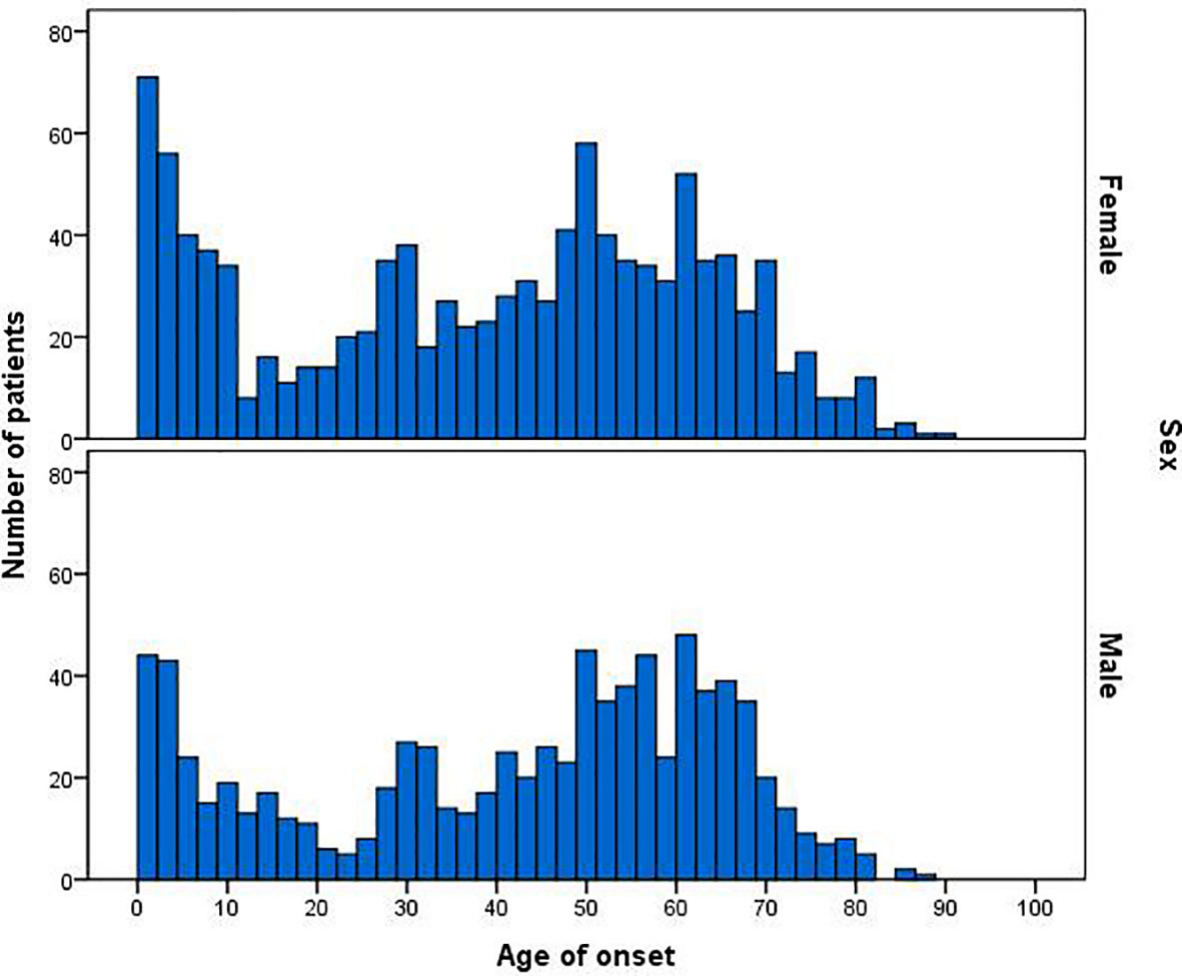
Age of onset in 1948 MG patients according to gender and age distribution.

Among the 1948 MG patients, 839 were males, and 1082 were females; the other 27 patients had no age and gender information. The male-to-female ratio was 1:1.28. The age of onset was from 1 month to 89 years old. As shown in **Figure 5**, the age of onset in MG patients had three peaks (first peak, 0–10 years old for both male and female patients; second peak, approximately 30 years old for female patients only; third peak, 50–70 years old for both male and female patients). Among the 1948 MG patients, 25.1% (484/1921, onset age < 19 years) were adolescents, with a male-to-female ratio of 1:1.44 (198/286). Early-onset MG (EOMG, onset age < 50 years) accounted for 56.8% of cases (1092/1921), with a male-to-female ratio of 1:1.49 (438/654). Late-onset MG (LOMG, onset age ≥ 50 years) accounted for 43.2% of cases (829/1921), with a male-to-female ratio of 1:1.07 (400/429). A difference in the sex ratio was apparent between the different age groups. While the average age of male patients was 41.83 ± 22.97 (n = 839), the average age of female patients was only 38.72 ± 23.62 (n = 1082), and this difference was significant (P < 0.01).

As shown in **Table 1**, the male-to-female ratio of Agrin-MG patients was 1:0.64 (11/7). While the majority of Agrin-MG patients were male, the majority of patients in the other antibody subgroups were female. MuSK-MG was the group with the smallest male-to-female ratio of 1:4. This was followed by LRP4, with a male-to-female ratio of 1:1.5. Finally, AChR-MG and TSN-MG both had a similar male-to-female ratio. In our sample, the average age of Agrin-MG is older than the average age of other antibody-positive subgroups. Among the 18 cases of Agrin-MG, 15 were older than 40 years, accounting for 83.33% of the total. The average age of onset of Agrin-MG and MuSK-MG were similar; hence, there were no significant differences (*P* > 0.05). Compared with the other antibody subgroups, Agrin-MG cases comprised more middle-aged and elderly patients and a higher proportion of men.

**Table 1:** Age and sex distribution of MG serological subgroups.

	Agrin-MG (18/18)	AChR-MG (1365/1392)	MuSK-MG (50/50)	LRP4-MG (15/15)	TSN-MG (466/466)	Total (1921/1948)
Sex						
Male	11	591	10	6	216	839
Female	7	774	40	9	250	1082
M:F	1: 0.64	1: 1.30	1: 4.00	1: 1.50	1: 1.16	1: 1.28
Age of onset	51.44 ± 21.66	40.89 ± 23.28	50.51 ± 14.52	28.87 ± 24.67	35.69 ± 23.63	40.08 ± 23.39

### 3.4 Analysis of Agrin-Ab Positive Only Patients Characteristics

We detected nine patients who were agrin antibody positive only, accounting for 1.82% (9/493) of TSN-MG patients and 0.46% (9/1948) of all patients with MG. The age of onset was less than 1 year old or more than 40 years old, and the male-to-female ratio was 1:1.25. Up to 88.9% (8/9) of the patients presented eye muscle weakness, and some presented limb-girdle muscle or bulbar muscle weakness. As shown in **Table 2**, the first episode in seven patients (77.84%) involved the ocular muscles, while the first episode in two patients (22.2%) involved the limb-girdle muscles. There were no patients where the first episode involved the bulbar muscle or cervical muscle. One patient was prone to fatigue, and a thymoma was found during physical examination. The pathological type was type B1, and this was completely relieved after thymectomy. The remaining seven patients presented a normal thymus (no thymus pathological data was available in one patient).

**Table 2:** Clinical data of only Agrin antibody-positive MG patients.

Patient code	Sex	Onset age	Course (year)	Initial symptom	Involved muscle	Neostigmine test	RNS	MGFA	ELISA		CBA (Score)			Treatment	PIS	Thymic pathology
									AChR	Titin	MuSK	LRP4	Agrin			
1	F	0.1	4	ocular	ocular	+	ND	I	**-**	**-**	**-**	**-**	+ (3)	Pyr (240-60mg/d) PSL (30-5 mg/d)	CR	Normal
2	F	0.8	6	ocular	ocular	+	ND	I	**-**	**-**	**-**	**-**	+ (2)	Pyr (240-60 mg/d) PSL (30-10 mg/d)	CR	Normal
3	F	58	2	ocular	ocular	+	–	I	**-**	**-**	**-**	**-**	+ (3)	Pyr (120-60 mg/d) PSL(30-15 mg/d)	CR	Normal
4	F	60	6	ocular	ocular	–	+	I	**-**	**-**	**-**	**-**	+ (4)	Pyr(240 mg/d)	Unchanged	Normal
5	F	73	4	ocular	Ocular; limb	+	ND	IIa	**-**	**-**	**-**	**-**	+ (3)	Pyr(240 mg/d)	CR	Normal
6	M	59	1	limb	Ocular; bulbar; limb	+	+	IVa	**-**	**-**	**-**	**-**	+ (4)	Pyr (240 mg/d) PSL (60-30 mg/d)	R	Normal
7	M	44	1	limb	limb	+	ND	IIa	**-**	**-**	**-**	**-**	+ (4)	TX	CR	Thymoma type B1
8	M	59	1	ocular	Ocular; bulbar	+	–	IIIb	**-**	**-**	**-**	**-**	+ (4)	PSL (20-10 mg/d)	Unchanged	Normal
9	M	67	2.5	ocular	Ocular; limb	/	/	IVb	**-**	**-**	**-**	**-**	+ (4)	/	/	/

Pyr, Pyridostigmine; PSL, Prednisolone; PIS, postintervention status. MM, minimal manifestations; ND, not determined.

### 3.5 Analysis of Agrin/AChR-MG Patients Characteristics

We detected nine cases of agrin antibody-positive and AChR antibody-positive patient serum (called Agrin/AChR-MG), accounting for 0.46% (9/1948) of all patients with MG. The nine patients had a male-to-female ratio of 3.5 to 1, and all males were middle-aged or elderly men older than 40 years old. As shown in **Table 3**, of the seven Agrin/AChR-MG patients with clinical data, six patients (85.71%) had the first episode in the ocular muscle, and one patient had the first episode in bulbar muscle. All of the seven patients presented ocular muscle weakness, and some presented bulbar muscle or limb-girdle muscle weakness. The clinical symptoms were moderate to severe generalized or mild ophthalmic muscle weakness. In total, three cases were positive for Titin antibody, including two elderly patients and one middle-aged male patient, suggesting that the patients may be accompanied by thymoma, but it has not been confirmed by imaging and pathology. Interestingly, serum Titin-Ab was negative in both patients with type B2 thymoma.

**Table 3:** Clinical data of Agrin/AChR double positive MG patients.

Patient code	Sex	Onset age	Cours (year)	Initial symptom	Involve muscle	Neostigmine test	RNS	MGFA	ELISA		CBA (Score)			Treatment	PIS	Thymus pathology
									AChR	Titin	MuSK	Lrp4	Agrin			
10	F	35	1	ocular	Ocular; bulbar	+	+	IIIb	+	**-**	**-**	**-**	+ (3)	Pyr (240-270 mg/d) PSL (30-15 mg/d) AZA (100 mg/d) TX	R	Thymoma type B2
11	F	73	1.5	ocular	Ocular; Bulbar; limb	+	ND	IVb	+	+	**-**	**-**	+ (4)	Pyr (240 mg/d) PSL (60-0 mg/d) Tac (1 mg/d)	CR	normal
12	M	42	3	ocular	ocular	+	–	I	+	+	**-**	**-**	+ (4)	Pyr (240 mg/d) PSL (60-40 mg/d)	CR	normal
13	M	43	3	ocular	Ocular; bulbar	+	+	IIIb	+	**-**	**-**	**-**	+ (3)	Pyr (240-60 mg/d) PSL (20-5 mg/d) AZA (100-50 mg/d) TX	R	Thymoma type B2
14	M	62	2	ocular	ocular	+	ND	I	+	**-**	**-**	**-**	+ (3)	Pyr (180-60 mg/d) PSL (50-10 mg/d)	R	normal
15	M	63	3	ocular	ocular	+	ND	I	+	**-**	**-**	**-**	+ (4)	Pyr (180 mg/d) PSL (30 mg/d)	CR	normal
16	M	66	1	bulbar	Ocular; bulbar	/	/	IIIb	+	+	**-**	**-**	+ (4)	/	/	/
17	M	49	4	/	/			/	+	**-**	**-**	**-**	+ (3)	/	/	/
18	M	72	3	/	/			/	+	**-**	**-**	**-**	+ (4)	/	/	/

Pyr, Pyridostigmine; PSL, Prednisolone; AZA, azathioprine; Tac, Tacrolimus; ND, not determined.

/, No information; CR, complete remission (no medication); R, pharmacologic remission.

In comparison with MG patients who were only agrin antibody positive, Agrin/AChR-MG patients demonstrated a higher male-to-female ratio. The cases were more likely to involve the bulbar muscle. A greater proportion of the patients had an MGFA score of III or higher and presented more severe clinical symptoms.

### 3.6 Comparison of Agrin-MG With Other Antibody-Positive MG

In most cases, the first Agrin-MG episode involved the ocular muscles, although the bulbar muscles and limb-band muscles were often involved, especially in cases showing more severe symptoms. In contrast with the other antibody subgroups (as shown in **Table 4**), Agrin-MG was more common in middle-aged and elderly men, with a male-to-female ratio of 1:0.64. Patients in this group were also likely to present a thymoma, although the prognosis was better. The subtype of agrin antibodies was IgG1 and IgG3 (as shown in **Supplementary Figure 6**). The relationship between antibodies and clinical disease severity remains unclear.

**Table 4:** Comparison of characteristics of MG serology subgroups.

	Agrin	AChR			Clustered AChR	MuSK	LRP4	SNMG
		Early onset	Late onset	Thymoma type				
Percentage of patients	1-9	15-25	35–45	10-15	5% of SNMG	1–10	1-5	10-20
Age (year)	Any	<50	≥50	Any	Any	Any	Any	Any
Sex (M:F)	1:0.64	1: 1.6	1: 1	1: 1.2	–	1: 3	1: 1.5	1: 1.2
Involved muscle	Ocular;Bulbar; Limb	Ocular;Bulbar; Limb			Ocular;Bulbar; Limb	Bulbar; Respiratory	Ocular; Limb	Ocular;Bulbar; Limb
Thymic pathology	Normal or thymoma	Hyperplasia	Atrophy or normal	Type AB or type B	Variable	Normal	Normal	Normal or hyperplasia
Isotypes	IgG1, IgG3	IgG1,IgG2,IgG3			IgG1	IgG4	IgG1,IgG2,IgG3	–
Correlation of antibody titre with disease grade	Unclear	No			–	Yes	Unclear	–
Representative references	(18, 20, 25)	(6, 7, 22, 23)			(21, 26)	(8–10, 24, 27)	(11–13, 28)	(16, 17)

-, No data at the moment.

AChR-MG is divided into three types, early-onset (EOMG), late-onset (LOMG), and thymoma (27, 28). EOMG refers to patients whose age of onset is less than 50 years old, and the male-to-female ratio was 1:1.5. In most cases, the thymus was hyperplastic, and the response to thymectomy was good. LOMG refers to patients whose age of onset is greater than or equal to 50 years old, with a male-to-female ratio of 1:1. In most cases, the thymus was atrophied or normal, and there was often no response to thymectomy. Thymoma-type MG patients are often double-positive for AChR-Ab and Titin-Ab. The pathological type of thymoma is primarily type AB or type B (28). The male-to-female ratio was 1:1. The subtypes of AChR antibodies were mostly IgG1 and IgG2. AChR antibody levels do not show a correlation with disease severity (29, 30).

MuSK-MG presents a high ratio of bulbar muscle to respiratory muscle involvement, and the male-to-female ratio was 1:1.5. No pathological changes of the thymus were reported in these patients, and no response to thymectomy was reported. IgG4 antibodies against MuSK play an important role in pathogenesis, and antibody titer is usually positively correlated with disease severity (31, 32). In LRP4-MG patients, the clinical symptoms are relatively mild, and most of these patients have mild ocular or systemic muscle weakness. Most of the LRP4-MG patients were female with a normal thymus. LPR4 antibodies were mostly IgG1 subtype, and the relationship between antibody levels and clinical symptoms is unclear (23, 33).

Low-affinity antibodies are also pathogenic in the body, and clustered AChR-Ab can be detected in about 5% of AChR-Ab-negative patients using more sensitive cell-based analysis. Most of these antibodies are IgG1 subtypes (34). The clinical manifestations are similar to those of AChR-MG. In 10–20% of MG cases, patients are antibody-negative, although these cases may involve uncharacterized pathogenic antibodies against unknown antigens in the postsynaptic membrane. The male-to-female ratio in these patients is 1:1, and the thymus is mostly normal or hyperplastic (35). On the whole, the clinical characteristics of MG patients with different antibodies are different. In most cases, the ocular muscles are the starting site, although the bulbar or limb-girdle muscles can also be the starting site.

### 3.7 Treatment and Prognosis of Agrin-MG Patients

The most commonly used drugs for patients with MG are cholinesterase inhibitors (pyridostigmine) and corticosteroids (prednisone). Treatment and prognosis information was available for eight agrin-positive only patients (doses shown in **Table 2**). Two patients were treated with pyridostigmine alone, and the drug was effective in one patient and ineffective in the other. One patient was treated with prednisone alone, and no improvement was evident. The clinical symptoms of four patients were treated with pyridostigmine combined with prednisone, and this treatment combination proved effective. In one patient with thymoma (who did not receive any drug treatment), the ease of fatigue was completely alleviated after thymectomy.

Treatment and prognosis information was available for six cases of Agrin/AChR-MG, including three cases that only involved the eye muscles and three cases presenting moderate or severe generalized myasthenia. These patients were treated with pyridostigmine and prednisone combination treatment. In several of these patients, this treatment was combined with azathioprine or tacrolimus (dose shown in **Table 3**), and the symptoms were well controlled. These results suggest that treatment with cholinesterase inhibitor only or corticosteroid only is not effective in agrin-positive patients. Therefore, these treatments should be used in combination, and immunosuppressants should be added where necessary.

All three patients presenting with thymoma underwent thymectomy. Thymus resection involved either thoracoscopy (one case) or traditional thoracotomy (two cases). The symptoms were all relieved effectively. Thymectomy is recommended for patients with thymoma or Titin antibody positivity.

## 4 Discussion

This study reports the demographic and clinical characterization of MG patients with pathogenic agrin antibodies (Agrin-Ab) in a Chinese population using a newly developed CBA of full-length agrin. This analysis indicated that 18 Agrin-MG patients (among a total of 1948 MG patients) were detected. Nine patients were Agrin-Ab positive only, and nine patients were AChR/Agrin-Ab double-positive. Ocular weakness was the most common initial symptom in these patients, and a combination of acetylcholinesterase inhibitor and prednisone was effective in managing MG symptoms. No agrin antibody was found in 286 control samples, all of them were negative. This study provides important prevalence data for Agrin-Ab in a Chinese population with MG and valuable clinical guidance for treatment of such cases.

Studies have revealed that mice immunized with N-agrin demonstrate muscle weakness and exhibit symptoms similar to human MG (13). Previous studies have detected anti-agrin antibodies in the serum of MG patients (14, 36), and these antibodies can inhibit MuSK phosphorylation and AChR cluster. However, how agrin antibody causes MG is not entirely clear. The mechanism may be similar to AChR-Ab, which causes structural destruction through complement activation at the NMJ. Consistent with this hypothesis, most of the agrin antibodies detected in the serum of Agrin-MG patients belong to the IgG1 and IgG3 subclass. Agrin is anchored to the basal lamina through the N-terminal laminin binding domain, and the C-terminal binds to Lrp4, which induces the dimerization and autoactivation of MuSK. Agrin antibodies binding to C-terminal could block the Lrp4 binding, thereby inhibiting MuSK activation and AChR clustering, preventing NMJ formation. The underlying pathogenic mechanism of Agrin-Ab binding to the N-terminal must be investigated. In previous studies, the sera of nine (out of 65) Amyotrophic Lateral Sclerosis patients were agrin antibody positive (37). The importance of agrin antibodies in other neuroimmune diseases remains to be investigated.

Recent research used Mini-Agrin ELISA and CBA methods to detect five agrin antibody-positive cases in 54 cases of MG patients (36) (9.2%). The five cases of Agrin-MG had MGFA grades ranging from IIa to V. All patients had normal thymus, and no thymectomy was performed. In another study using an ELISA method to detect the C-terminus of agrin, seven agrin antibody-positive cases were identified in 93 MG patients (14) (7.5%). In the present study, we used a CBA method targeting the human full-length N-agrin protein. In total, we tested 1948 MG patient samples from 26 provinces in China and found that the agrin antibody positive rate in Chinese MG patients was only 0.92%.

Another study found 26 Agrin-MG patients among 181 cases of DNMG (15), an incidence rate of 14.36%, of which 23 cases were also LRP4 antibody-positive. In comparison with the antibody-negative patients, agrin antibody-positive and LRP4 antibody-positive patients showed systemic symptoms. Thus, 70% were MGFA III–V patients, far higher than the 39% incidence observed in antibody-negative patients. Among our TSN-MG patients, the agrin antibody positive rate was 1.82% (9/493), including five cases with an MGFA classification of II–IVb and four cases involving only the ocular muscles (which did not develop to the generalized type). Clinical data were available for seven Agrin/AChR-MG patients, and the MGFA classification was IIIb or IVb in four cases (57.14%). These patients were more likely to present bulbar muscle weakness, and the prognosis was more severe than for agrin antibody positive only cases. Some Agrin-MG cases were accompanied by thymoma in our cohort, and these patients were successfully treated with a thymectomy.

We used a CBA method targeting the full-length human agrin protein, which has a decisive effect on the serological typing of MG, especially the detection of triple-seronegative MG patients. In comparison with ELISA and Mini-Agrin CBA, the full-length agrin protein expressed by CBA has a natural conformation (closer to the original state of the human protein) and contains more spatial epitopes. Studies comparing the sensitivities of CBA and ELISA have reported that the sensitivity of CBA is higher than that of ELISA (21, 26, 38). Live CBAs have natural epitopes and higher specificity. By contrast, during the cross-linking of molecules induced by fixing of the cells, epitopes may be newly introduced or destroyed. Since agrin is a secretory protein, thus not expressed at the cell surface, we depend on the use of fixed cell-based assays and staining of intracellular antigen.

This study provides essential prevalence data for Agrin-MG in a Chinese population and valuable clinical guidance for treating such cases. In this study, due to the small number of Agrin-MG patients, the clinical characteristics of these patients and the guidance of clinical medication need more in-depth research. Although the traditional combination therapy of pyridostigmine and prednisone can effectively control the disease of Agrin-MG patients, due to these patients are older, hormone use should be cautious, and there is still a need for more accurate medication research in this subgroup of patients.

## Data Availability Statement

The original contributions presented in the study are included in the article/**Supplementary Material**. Further inquiries can be directed to the corresponding authors.

## Author Contributions

Design and conceptualized study, SW, HY, and FG. Drafted the manuscript, SW. Major role in the acquisition of data, analyzed and interpreted the data, RG, LW, YNZ, JL, XZ, JZ, HF, QZ, YKZ, JY, XC, PG, and TC. Revised the manuscript for intellectual content, FG and TC. All authors contributed to the article and approved the submitted version.

## Funding

This work was supported by the Key Science and Technology Project of Henan Province (grant number 182102311171/202102310402); Key Research Projects of Henan Higher Education Institutions (grant numbers 19A320045); Project of Basic Research Fund of Henan Institute of Medical and Pharmaceutical Sciences (grant number 2020SP0102 2020BP0112 2020BP0117 2020BP0201); and Special Project of Henan Province Traditional Chinese Medicine Scientific Research (grant number 20-21ZY1044).

## Conflict of Interest

The authors declare that the research was conducted in the absence of any commercial or financial relationships that could be construed as a potential conflict of interest.

## Publisher’s Note

All claims expressed in this article are solely those of the authors and do not necessarily represent those of their affiliated organizations, or those of the publisher, the editors and the reviewers. Any product that may be evaluated in this article, or claim that may be made by its manufacturer, is not guaranteed or endorsed by the publisher.
