# Supplementary material for: Antibodies to Full-Length Agrin Protein in Chinese Patients With Myasthenia Gravis
Source: Front Immunol. 2021 Dec 8;12:753247. doi: 10.3389/fimmu.2021.753247 (PMC8692888; doi:10.3389/fimmu.2021.753247)
Supplement: Supplementary file 1 [file DataSheet_1.pdf]

Supplementary Material

1 Supplementary Figures and Tables

1.1 Supplementary Figures

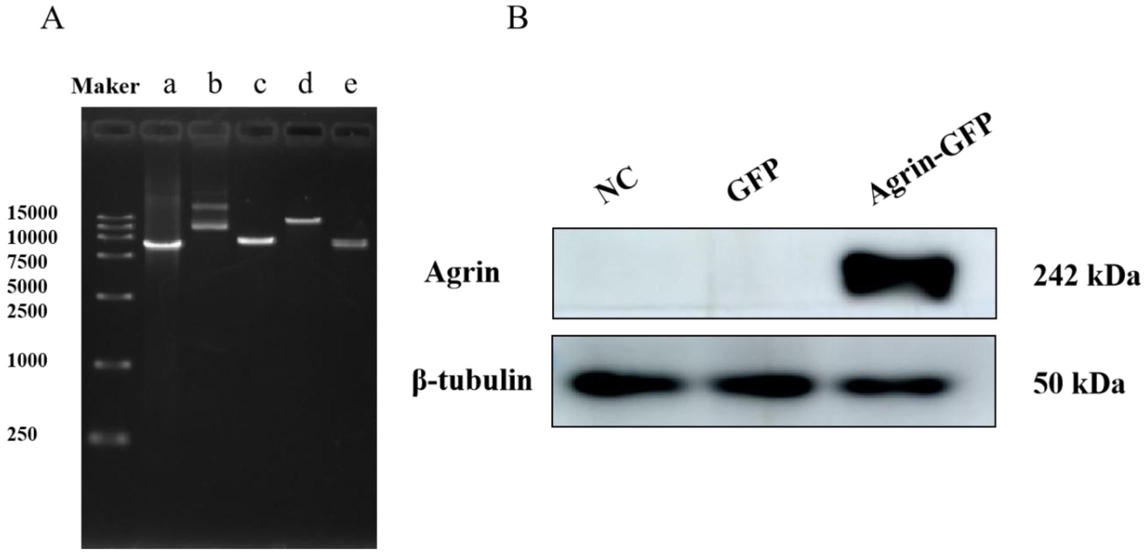

Supplementary Figure 1. Verification of Agrin plasmid and eukaryotic-expressed protein. A. Enzymatic identification of Agrin plasmid. a, amplified Agrin gene (6.2 kb); b, uncut plasmid construct encoding agrin; c, double digestion product of the empty pCMV-AC-GFP vector(6.6 kb); d, single digestion product of plasmid construct encoding agrin; e, double digestion product of plasmid construct encoding agrin. B. Western blot analysis of the eukaryotic expression of agrin; the size of the agrin-GFP fusion protein is approximately 242 kDa.

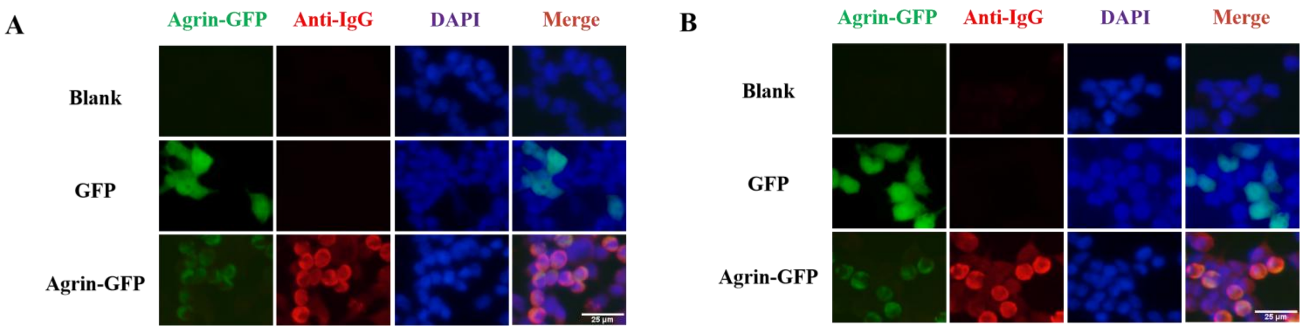

Supplementary Figure 2. Illustration of antibody specificity. A.Incubate cells with positive serum. B.Incubate cells with commercial antibodies. Blank: No plasmids have been transfected; GFP: Cells Transfected with pCMV6-AC-GFP vector, it can express GFP protein; Agrin-GFP: Cells Transfected with pCMV6-AC-Agrin-GFP, it can express Agrin-GFP fusion protein.

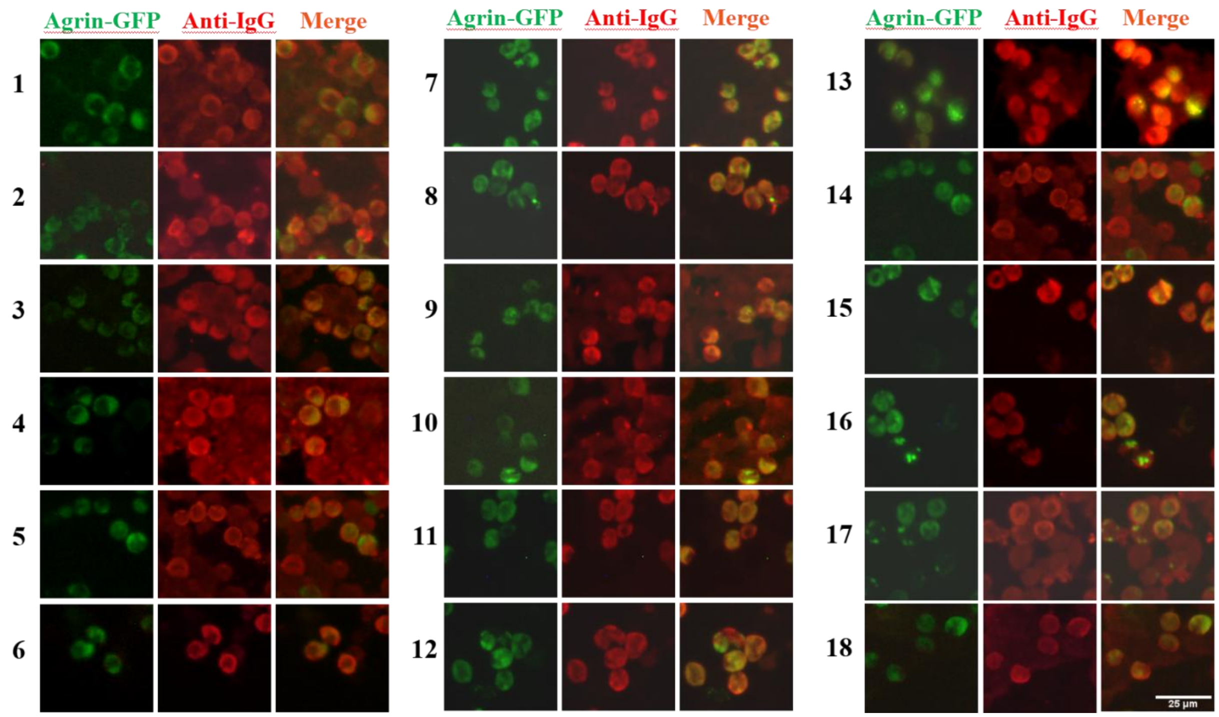

Supplementary Figure 3. The results for 18 Agrin positive samples. From 1 to 18 respectively represent 18 Agrin-MG patients.

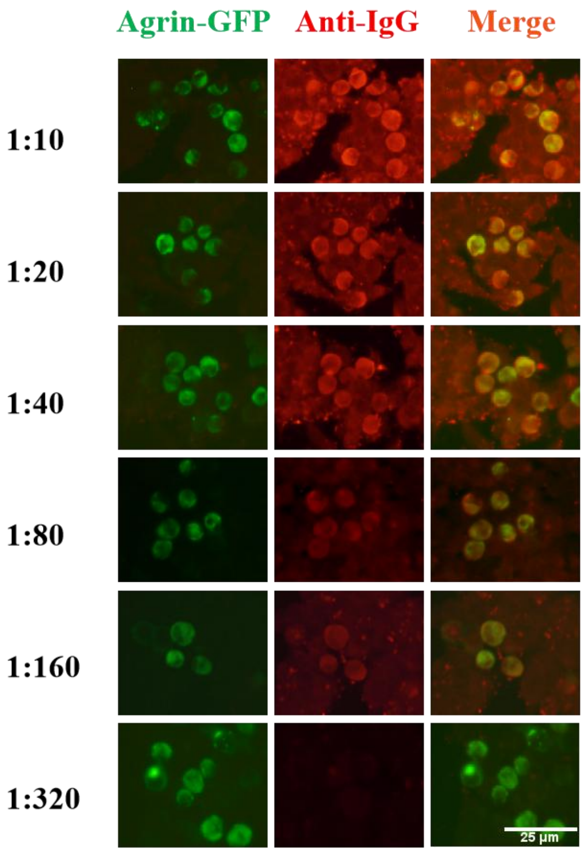

Supplementary Figure 4. Immunofluorescence images of different dilutions of serum. The serum was diluted to different degrees, and the results showed that Agrin-CBA diluted 1:160 can also be positive.

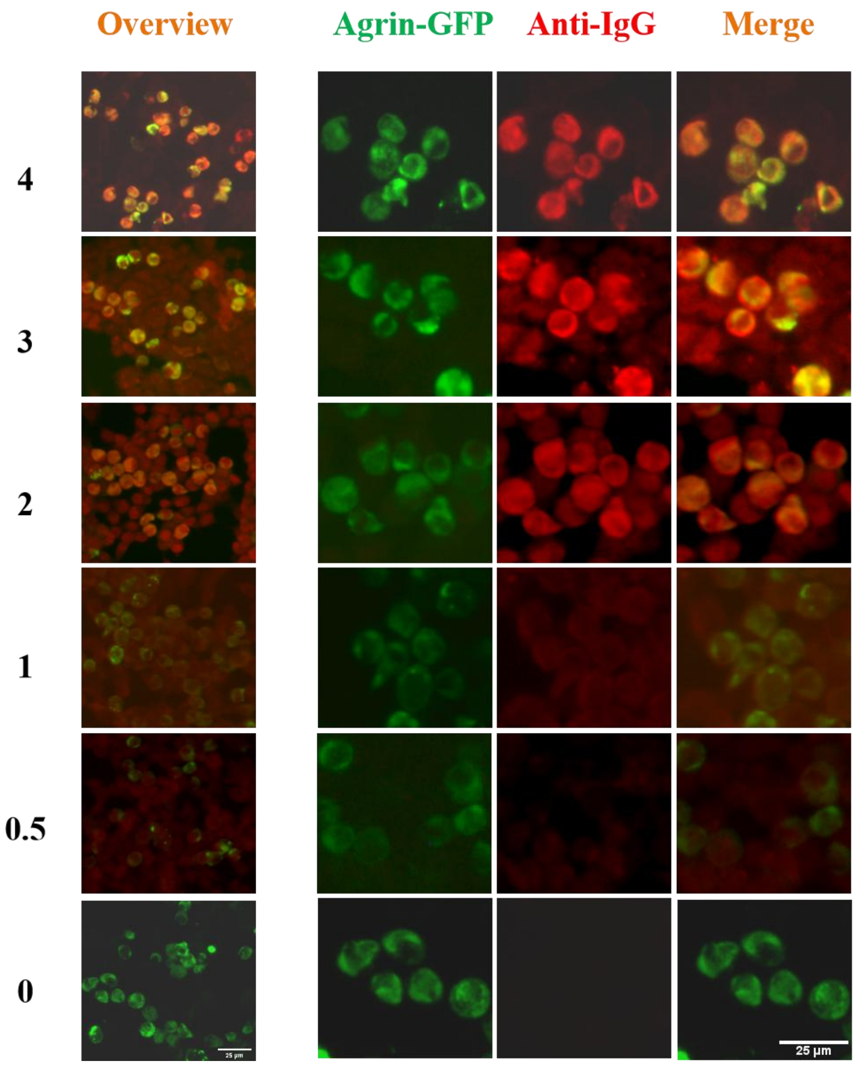

Supplementary Figure 5. The representative images of the CBA in each level of visual scores. 0, no signal; 0.5 for very weak labeling of a few cells with no definite colocalization; 1, 1 for weak labeling of some cells with colocalization,; 2 for labeling of 20–50% of cells with accurate colocalization, 3 for labeling of 50–80% of cells with perfect colocalization, and 4 for labeling of all transduced cells showing perfect colocalization.

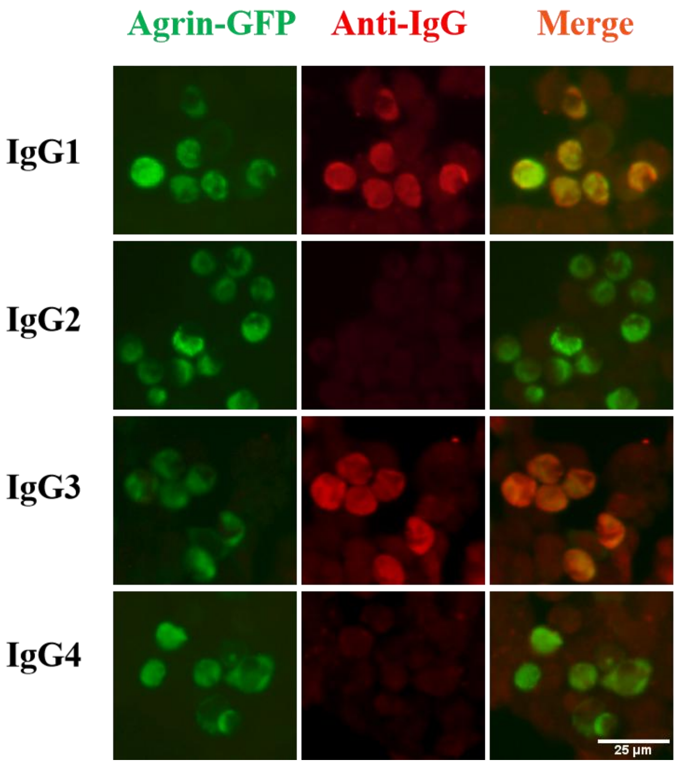

Supplementary Figure 6. Representative plots for the IgG subclass determination. Using mouse anti-human IgG1-4 antibodies and Alexa Fluor-594 donkey anti-mouse IgG for cell-based assays, the results showed that the IgG in the serum of Agrin-MG patients was IgG1 and IgG3.

1.2 Supplementary Tables

Supplementary Table 1. Population distribution of patients with MG from different provinces/cities in China

Province	Number of MG Patients	Percentage (%)	Number of Agrin-MG
Anhui	23	1.18	0
Beijing	19	0.98	0
Chongqing	31	1.59	1
Fujian	34	1.75	1
Guangdong	56	2.87	0

Guangxi	11	0.56	0
Gansu	25	1.28	0
Henan	1005	51.59	12
Hebei	16	0.82	0
Heilongjiang	46	2.36	0
Hunan	12	0.62	0
Hubei	95	4.88	2
Inner Mongolia	7	0.36	0
Jiangsu	25	1.28	0
Jilin	17	0.87	0
Jiangxi	21	1.08	0
Ningxia	43	2.21	0
Shaanxi	243	12.47	1
Shanxi	73	3.75	0
Sichuan	41	2.1	1
Shanghai	16	0.82	0
Shandong	15	0.77	0
Tianjin	5	0.26	0
Xinjiang	30	1.54	0
Yunnan	15	0.77	0

Zhejiang	24	1.23	0

Supplementary Table 2. Age and sex distribution of MG serological subgroups

	Agrin-MG (18/18)	AChR-MG (1365/1392)	MuSK-MG (50/50)	LRP4-MG (15/15)	SN-MG (466/466)	Total (1921/1948)
Sex						
Male	11	591	10	6	216	839
Female	7	774	40	9	250	1082
M:F	1 : 0.64	1 : 1.30	1 : 4.00	1 : 1.50	1 : 1.16	1 : 1.28
Age of onset	51.44±21.66	40.89±23.28	50.51±14.52	28.87±24.67	35.69±23.63	40.08±23.39

1.3 Agrin Plasmid Information

NCBI Reference Sequence: NM_001305275.2

CDS Sequence : The red part is the signal peptide.

atggccg

61 gccggtccca cccgggcccg ctgcggccgc tgctgccgct ccttgtggtg gccgcgtgcg

121 tcctgcccgg agccggcggg acatgcccgg agcgcgcgct ggagcggcgc gaggaggagg

181 cgaacgtggt gctcaccggg acggtggagg agatcctcaa cgtggacccg gtgcagcaca

241 cgtactcctg caaggttcgg gtctggcggt acttgaaggg caaagacctg gtggcccggg

301 agagcctgct ggacggcggc aacaaggtgg tgatcagcgg ctttggagac cccctcatct

361 gtgacaacca ggtgtccact ggggacacca ggatcttctt tgtgaaccct gcacccccat

421 acctgtggcc agcccacaag aacgagctga tgctcaactc cagcctcatg cggatcaccc

481 tgcggaacct ggaggaggtg gagttctgtg tggaagataa acccgggacc cacttcactc

541 cagtgcctcc gacgcctcct gatgcgtgcc ggggaatgct gtgcggcttc ggcgccgtgt

601 gcgagcccaa cgcggagggg ccgggccggg cgtcctgcgt ctgcaagaag agcccgtgcc

661 ccagcgtggt ggcgcctgtg tgtgggtcgg acgcctccac ctacagcaac gaatgcgagc

721 tgcagcgggc gcagtgcagc cagcagcgcc gcatccgcct gctcagccgc gggccgtgcg

781 gctcgcggga cccctgctcc aacgtgacct gcagcttcgg cagcacctgt gcgcgctcgg

841 ccgacgggct gacggcctcg tgcctgtgcc ccgcgacctg ccgtggcgcc cccgagggga

901 ccgtctgcgg cagcgacggc gccgactacc ccggcgagtg ccagctcctg cgccgcgcct

961 gcgcccgcca ggagaatgtc ttcaagaagt tcgacggccc ttgtgacccc tgtcagggcg

1021 ccctccctga cccgagccgc agctgccgtg tgaacccgcg cacgcggcgc cctgagatgc

1081 tcctacggcc cgagagctgc cctgcccggc aggcgccagt gtgtggggac gacggagtca

1141 cctacgaaaa cgactgtgtc atgggccgat cgggggccgc ccggggtctc ctcctgcaga

1201 aagtgcgctc cggccagtgc cagggtcgag accagtgccc ggagccctgc cggttcaatg

1261 ccgtgtgcct gtcccgccgt ggccgtcccc gctgctcctg cgaccgcgtc acctgtgacg

1321 gggcctacag gcccgtgtgt gcccaggacg ggcgcacgta tgacagtgat tgctggcggc

1381 agcaggctga gtgccggcag cagcgtgcca tccccagcaa gcaccagggc ccgtgtgacc

1441 aggccccgtc cccatgcctc ggggtgcagt gtgcatttgg ggcgacgtgt gctgtgaaga

1501 acgggcaggc agcgtgtgaa tgcctgcagg cgtgctcgag cctctacgat cctgtgtgcg

1561 gcagcgacgg cgtcacatac ggcagcgcgt gcgagctgga ggccacggcc tgtaccctcg

1621 ggcgggagat ccaggtggcg cgcaaaggac cctgtgaccg ctgcgggcag tgccgctttg

1681 gagccctgtg cgaggccgag accgggcgct gcgtgtgccc ctctgaatgc gtggctttgg

1741 cccagcccgt gtgtggctcc gacgggcaca cgtaccccag cgagtgcatg ctgcacgtgc

1801 acgcctgcac acaccagatc agcctgcacg tggcctcagc tggaccctgt gagacctgtg

1861 gagatgccgt gtgtgctttt ggggctgtgt gctccgcagg gcagtgtgtg tgtccccggt

1921 gtgagcaccc cccgcccggc cccgtgtgtg gcagcgacgg tgtcacctac ggcagtgcct

1981 gcgagctacg ggaagccgcc tgcctccagc agacacagat cgaggaggcc cgggcagggc

2041 cgtgcgagca ggccgagtgc ggttccggag gctctggctc tggggaggac ggtgactgtg

2101 agcaggagct gtgccggcag cgcggtggca tctgggacga ggactcggag gacgggccgt

2161 gtgtctgtga cttcagctgc cagagtgtcc caggcagccc ggtgtgcggc tcagatgggg

2221 tcacctacag caccgagtgt gagctgaaga aggccaggtg tgagtcacag cgagggctct

2281 acgtagcggc ccagggagcc tgccgaggcc ccaccttcgc cccgctgccg cctgtggccc

2341 ccttacactg tgcccagacg ccctacggct gctgccagga caatatcacc gcagcccggg

2401 gcgtgggcct ggctggctgc cccagtgcct gccagtgcaa cccccatggc tcttacggcg

2461 gcacctgtga cccagccaca ggccagtgct cctgccgccc aggtgtgggg ggcctcaggt

2521 gtgaccgctg tgagcctggc ttctggaact ttcgaggcat cgtcaccgat ggccggagtg

2581 gctgtacacc ctgcagctgt gatccccaag gcgccgtgcg ggatgactgt gagcagatga

2641 cggggctgtg ctcgtgtaag cccggggtgg ctggacccaa gtgtgggcag tgtccagacg

2701 gccgtgccct gggccccgcg ggctgtgaag ctgacgcttc tgcgcctgcg acctgtgcgg

2761 agatgcgctg tgagttcggt gcgcggtgcg tggaggagtc tggctcagcc cactgtgtct

2821 gcccgatgct cacctgtcca gaggccaacg ctaccaaggt ctgtgggtca gatggagtca

2881 catacggcaa cgagtgtcag ctgaagacca tcgcctgccg ccagggcctg caaatctcta

2941 tccagagcct gggcccgtgc caggaggctg ttgctcccag cactcacccg acatctgcct

3001 ccgtgactgt gaccacccca gggctcctcc tgagccaggc actgccggcc ccccccggcg

3061 ccctccccct ggctcccagc agtaccgcac acagccagac cacccctccg ccctcatcac

3121 gacctcggac cactgccagc gtccccagga ccaccgtgtg gcccgtgctg acggtgcccc

3181 ccacggcacc ctcccctgca cccagcctgg tggcgtccgc ctttggtgaa tctggcagca

3241 ctgatggaag cagcgatgag gaactgagcg gggaccagga ggccagtggg ggtggctctg

3301 gggggctcga gcccttggag ggcagcagcg tggccacccc tgggccacct gtcgagaggg

3361 cttcctgcta caactccgcg ttgggctgct gctctgatgg gaagacgccc tcgctggacg

3421 cagagggctc caactgcccc gccaccaagg tgttccaggg cgtcctggag ctggagggcg

3481 tcgagggcca ggagctgttc tacacgcccg agatggctga ccccaagtca gaactgttcg

3541 gggagacagc caggagcatt gagagcaccc tggacgacct cttccggaat tcagacgtca

3601 agaaggattt tcggagtgtc cgcttgcggg acctggggcc cggcaaatcc gtccgcgcca

3661 ttgtggatgt gcactttgac cccaccacag ccttcagggc acccgacgtg gcccgggccc

3721 tgctccggca gatccaggtg tccaggcgcc ggtccttggg ggtgaggcgg ccgctgcagg

3781 agcacgtgcg atttatggac tttgactggt ttcctgcgtt tatcacgggg gccacgtcag

3841 gagccattgc tgcgggagcc acggccagag ccaccactgc atcgcgcctg ccgtcctctg

3901 ctgtgacccc tcgggccccg caccccagtc acacaagcca gcccgttgcc aagaccacgg

3961 cagcccccac cacacgtcgg ccccccacca ctgcccccag ccgtgtgccc ggacgtcggc

4021 ccccggcccc ccagcagcct ccaaagccct gtgactcaca gccctgcttc cacgggggga

4081 cctgccagga ctgggcattg ggcgggggct tcacctgcag ctgcccggca ggcaggggag

4141 gcgccgtctg tgagaaggtg cttggcgccc ctgtgccggc cttcgagggc cgctccttcc

4201 tggccttccc cactctccgc gcctaccaca cgctgcgcct ggcactggaa ttccgggcgc

4261 tggagcctca ggggctgctg ctgtacaatg gcaacgcccg ggcaaggac ttcctggcat

4321 tggcgctgct agatggccgc gtgcagctca ggtttgacac aggttcgggg ccggcggtgc

4381 tgaccagtgc cgtgccggta gagccgggcc agtggcaccg cctggagctg tcccggcact

4441 ggcgccgggg caccctctcg gtggatggtg agacccctgt tctgggcgag agtcccagtg

4501 gcaccgacgg cctcaacctg gacacagacc tctttgtggg cggcgtaccc gaggaccagg

4561 ctgccgtggc gctggagcgg accttcgtgg gcgccggcct gagggggtgc atccgtttgc

4621 tggacgtcaa caaccagcgc ctggagcttg gcattgggcc gggggctgcc acccgaggct

4681 ctggcgtggg cgagtgcggg gaccacccct gcctgcccaa cccctgccat ggcggggccc

4741 catgccagaa cctggaggct ggaaggttcc attgccagtg cccgcccggc cgcgtcggac

4801 caacctgtgc cgatgagaag agcccctgcc agcccaaccc ctgccatggg gcggcgccct

4861 gccgtgtgct gcccgagggt ggtgctcagt gcgagtgccc cctggggcgt gagggcacct

4921 tctgccagac agcctcgggg caggacggct ctgggccctt cctggctgac ttcaacggct

4981 tctcccacct ggagctgaga ggcctgcaca cctttgcacg ggacctgggg gagaagatgg

5041 cgctggaggt cgtgttcctg gcacgaggcc ccagcggcct cctgctctac aacgggcaga

5101 agacggacgg caagggggac ttcgtgtcgc tggcactgcg ggaccgccgc ctggagttcc

5161 gctacgacct gggcaagggg gcagcggtca tcaggagcag ggagccagtc accctgggag

5221 cctggaccag ggtctcactg gagcgaaacg gccgcaaggg tgccctgcgt gtgggcgacg

5281 gcccccgtgt gttgggggag tccccgaaat cccgcaaggt tccgcacacc gtcctcaacc

5341 tgaaggagcc gctctacgta gggggcgctc ccgacttcag caagctggcc cgtgctgctg

5401 ccgtgtcctc tggcttcgac ggtgccatcc agctggtctc cctcggaggc cgccagctgc

5461 tgaccccgga gcacgtgctg cggcaggtgg acgtcacgtc ctttgcaggt cacccctgca

5521 cccgggcctc aggccacccc tgcctcaatg gggcctcctg cgtcccgagg gaggctgcct

5581 atgtgtgcct gtgtcccggg ggattctcag gaccgcactg cgagaagggg ctggtggaga

5641 agtcagcggg ggacgtggat accttggcct ttgacgggcg gacctttgtc gagtacctca

5701 acgctgtgac cgagagcgaa ctggccaatg agatccccgt ccccgaaact ctggattccg

5761 gggcccttca cagcgagaag gcactgcaga gcaaccactt tgaactgagc ctgcgcactg

5821 aggccacgca ggggctggtg ctctggagtg gcaaggccac ggagcgggca gactatgtgg

5881 cactggccat tgtggacggg cacctgcaac tgagctacaa cctgggctcc cagcccgtgg

5941 tgctgcgttc caccgtgccc gtcaacacca accgctggtt gcgggtcgtg gcacataggg

6001 agcagaggga aggttccctg caggtgggca atgaggcccc tgtgaccggc tcctccccgc

6061 tgggcgccac gcagctggac actgatggag ccctgtggct tgggggcctg ccggagctgc

6121 ccgtgggccc agcactgccc aaggcctacg gcacaggctt tgtgggctgc ttgcgggacg

6181 tggtggtggg ccggcacccg ctgcacctgc tggaggacgc cgtcaccaag ccagagctgc

6241 ggccctgccc caccccatga

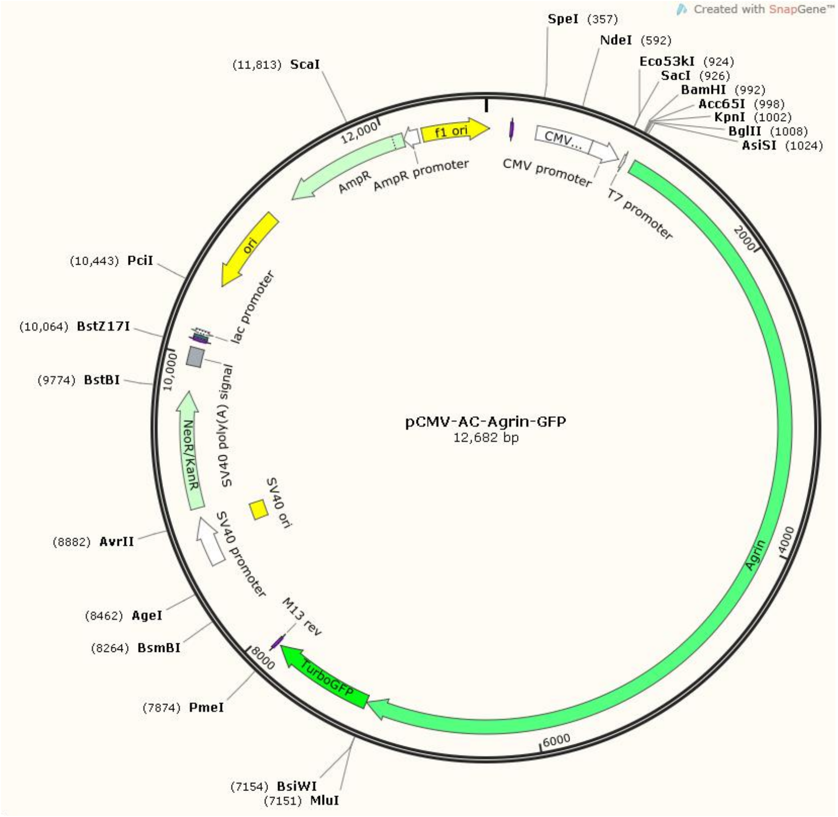
Created with SnapGene™
SpeI (357)
NdeI (592)
Eco53kI (924)
SacI (926)
BamHI (992)
Acc65I (998)
KpnI (1002)
BglII (1008)
AsiSI (1024)
(11,813) ScaI
(10,443) PciI
(10,064) BstZ17I
(9774) BstBI
(8882) AvrII
(8462) AgeI
(8264) BsmBI
(7874) PmeI
(7154) BsiWI
(7151) MluI
f1 ori
CMV...
CMV promoter
T7 promoter
AmpR
AmpR promoter
ori
lac promoter
SV40 poly(A) signal
NeoR/KanR
SV40 ori
SV40 promoter
M13 rev
TurboGFP
Agrin
12,000
10,000
8000
6000
4000
2000
pCMV-AC-Agrin-GFP
12,682 bp
